# Metabolic Regulation of Longevity and Immune Response in *Caenorhabditis elegans* by Ingestion of *Lacticaseibacillus rhamnosus* IDCC 3201 Using Multi-Omics Analysis

**DOI:** 10.4014/jmb.2402.02025

**Published:** 2024-04-03

**Authors:** Daniel Junpyo Lee, Ju Young Eor, Min-Jin Kwak, Junbeom Lee, An Na Kang, Daye Mun, Hyejin Choi, Minho Song, Jong Nam Kim, Jun-Mo Kim, Jungwoo Yang, Hyung Wook Kim, Sangnam Oh, Younghoon Kim

**Affiliations:** 1 Department of Agricultural Biotechnology and Research Institute of Agriculture and Life Science, Seoul National University, Seoul 08826, Rrepublic of Korea; 2 Department of Animal Science and Biotechnology, Chungnam National University, Daejeon 34134, Republic of Korea; 3 Department of Food Science & Nutrition, Dongseo University, Busan 47011, Republic of Korea; 4 Department of Animal Science and Technology, Chung-Ang University, Anseong 17546, Republic of Korea; 5 Department of Microbiology, College of Medicine, Dongguk University, Gyeongju 38066, Republic of Korea; 6 College of Life Sciences, Sejong University, Seoul 05006, Republic of Korea; 7 Department of Functional Food and Biotechnology, Jeonju University, Jeonju 55069, Republic of Korea

**Keywords:** *Lacticaseibacillus rhamnosus*, *C. elegans*, immune response, multi-omics analysis

## Abstract

Probiotics, specifically *Lacticaseibacillus rhamnosus*, have garnered attention for their potential health benefits. This study focuses on evaluating the probiotic properties of candidate probiotics *L. rhamnosus* IDCC 3201 (3201) using the *Caenorhabditis elegans* surrogate animal model, a well-established in vivo system for studying host–bacteria interactions. The adhesive ability to the host’s gastrointestinal tract is a crucial criterion for selecting potential probiotic bacteria. Our findings demonstrated that 3201 exhibits significantly higher adhesive capabilities compared with *Escherichia coli* OP50 (OP50), a standard laboratory food source for *C. elegans* and is comparable with the widely recognized probiotic *L. rhamnosus* GG (LGG). In lifespan assay, 3201 significantly increased the longevity of *C. elegans* compared with OP50. In addition, preconditioning with 3201 enhanced *C. elegans* immune response against four different foodborne pathogenic bacteria. To uncover the molecular basis of these effects, transcriptome analysis elucidated that 3201 modulates specific gene expression related to the innate immune response in *C. elegans*. C-type lectin-related genes and lysozyme-related genes, crucial components of the immune system, showed significant upregulation after feeding 3201 compared with OP50. These results suggested that preconditioning with 3201 may enhance the immune response against pathogens. Metabolome analysis revealed increased levels of fumaric acid and succinic acid, metabolites of the citric acid cycle, in *C. elegans* fed with 3201 compared with OP50. Furthermore, there was an increase in the levels of lactic acid, a well-known antimicrobial compound. This rise in lactic acid levels may have contributed to the robust defense mechanisms against pathogens. In conclusion, this study demonstrated the probiotic properties of the candidate probiotic *L. rhamnosus* IDCC 3201 by using multi-omics analysis.

## Introduction

Interest and demand for probiotics are increasing due to their long history of safe use in fermented products and the recognition of their profitable effect on gut and health [1]. According to the Food and Agriculture Organization and the World Health Organization, the current definition of probiotics is “Live microorganisms which confer a health benefit when administered in adequate amounts [2]. Probiotics are acknowledged for their beneficial effects on host health, including their ability to compete with pathogenic bacteria, produce antimicrobial compounds, enhance the integrity of the host’s mucosal barrier, and modulate the immune system [3-6]. *Lactobacilli* are characterized by non-spore-forming, non-motile, gram-positive, and rod-shaped bacteria. These bacteria are normally found in animals’ mouths and gastrointestinal tracts [7]. Within the gastrointestinal microbiota, strains belonging to *Lactobacillus* stand out as one of the most predominant groups and are widely recognized as the most commonly used probiotic bacteria [8]. *Lactobacillus rhamnosus*, which has recently been denominated as *Lacticaseibacillus rhamnosus*, is one of the health-promoting bacteria.

*Caenorhabditis elegans* is known as a surrogate animal model, which is a suitable model for studying microbe–host interaction. Using *C. elegans* as an experimental model offers several advantages, including its transparent body, short lifespan, ease of handling, simple genetics, and cost-effectiveness [9]. An abundance of genetic resources about *C. elegans* allows a detailed investigation of host–microbe interactions. Bacteria act as a dietary source for *C. elegans*, influencing its phenotype, including factors like lifespan and immune response [10]. Feeding bacteria directly to *C. elegans* makes it an ideal model for studying host–microbe interactions. This approach minimizes the impact of external nutrients on *C. elegans*, allowing for a more focused examination of these interactions [11].

The whole transcriptomic analysis is widely used to examine the change in multiple genetic pathways in *C. elegans* [12]. Previous research demonstrated that *C. elegans* serves as a suitable experimental model for investigating genetic pathways, providing valuable insights into the functional aspects of anti-aging and innate immunity [13]. Moreover, in previous studies using *C. elegans*, metabolomics was used to unveil the metabolites linked to novel biological mechanisms associated with longevity and innate immune response [14, 15].

Several studies in *C. elegans* have demonstrated the positive impact of feeding certain strains of *Lactobacillus* on both lifespan and immune response [16-18]. This study was conducted to assess the probiotic properties of candidate probiotic *L. rhamnosus* IDCC 3201 using multi-omics analysis.

## Material and Methods

### Bacterial and *C. elegans* Strains and Culture Conditions

*C. elegans* strain *fer-15;fem-1*, which is known as a strain that is unable to give birth at 25°C without alteration in the phenotype, was used in all experiments conducted in this study. *Escherichia coli* OP50 (referred to as OP50) was used as a negative control feed for *C. elegans*, and it was grown in Luria–Bertani (LB) broth medium (BD Biosciences, USA) at 37°C for 24 h with shaking (225 rpm). *L. rhamnosus* GG (referred to as LGG) was used as a positive control feed for *C. elegans*, and it was grown in De Man–Rogosa–Sharpe (MRS) broth medium (BD Biosciences, Sparks, MD, USA) and incubated at 37°C for 48 h. *L. rhamnosus* IDCC 3201 (referred to as 3201) was grown under the same media and conditions as LGG. Four foodborne pathogenic bacteria were grown with the following medium and time. The gram-negative bacteria, *E. coli* O157:H7 EDL933 (EDL933), was grown at 37°C for 24 h in LB broth medium (BD Biosciences). *Salmonella* Typhimurium SL1344 (SL1344) was grown at 37°C for 24 h in a nutrient broth medium (BD Biosciences, Sparks, MD, USA), whereas two gram-positive bacteria, *Staphylococcus aureus* Newman (Newman) and *Listeria monocytogenes* EGD-e (EGD-e), were grown at 37°C for 24 h in brain–heart infusion (BHI) broth medium (BD Biosciences).

### In vivo Adhesive Assay Using *C. elegans*

To evaluate whether 3201 can colonize in *C. elegans* intestine, an in vivo adhesive assay was conducted following published methods with slight modiﬁcations [16]. Briefly, worms were maintained on nematode growth medium (NGM) agar seeded with OP50. After the worms got eggs in their body, eggs were extracted using sodium hypochlorite–sodium hydroxide solution. Then synchronized L1 stage worms were grown on NGM agar seeded with OP50 until they reached the L4 stage at 25°C. The worms were transferred to NGM agar seeded with OP50 (8.0 × 10^9^ colony-forming unit [CFU/ml]), 3201 (8.0 × 10^9^ CFU/ml), or LGG (8.0 × 10^9^ CFU/ml), respectively, to evaluate the degree of bacterial colonization in the *C. elegans* intestinal tract. After 48 h conditioning period, 10 worms in each group were picked randomly and placed on BHI broth medium (BD Biosciences) agar plates for 5 min, which contain gentamycin (25 μg/ml). Next, worms were transferred to a 1.5 ml Eppendorf tube containing M9 buffer with Triton X-100 and were mechanically disrupted using a pestle (Kontes Glass, USA). The diluted worms were plated on an MRS broth medium (BD Biosciences) agar plate and incubated at 37°C for 48 h to reach the appropriate growth phase. The experiment involved 6 repetitions for each treatment, with 10 worms per repetition, resulting in a total of 60 worms used per treatment.

### Lifespan and Killing Assay

To evaluate whether 3201 influences *C. elegans*’ lifespan and immune response against foodborne pathogenic bacteria, we established methods with slight modifications based on a previous study [16, 17]. About the lifespan assay, synchronized L1 stage worms were placed on NGM agar seeded with OP50 until they reached the L4 stage at 25°C. Then *C. elegans* were individually transferred with a platinum wire onto 35-mm-diameter NGM agar plates seeded with OP50 (8.0 × 10^9^ CFU/ml), 3201 (8.0 × 10^9^ CFU/ml), or LGG (8.0 × 10^9^ CFU/ml), respectively. For lifespan assay, 100 *C. elegans* per treatment were used in five plates (20 worms per plate). The numbers of live *C. elegans* were counted every day and transferred to a new plate every 2 days. To determine whether the worms were dead or alive, they were gently touched with a platinum wire. The assay was conducted until all *C. elegans* died.

About the killing assay, L4 stage *C. elegans* were placed onto 35-mm-diameter NGM agar plates seeded with OP50 (8.0 × 10^9^ CFU/ml), 3201 (8.0 × 10^9^ CFU/ml), or LGG (8.0 × 10^9^ CFU/ml), respectively. After the 48-h preconditioning, *C. elegans* were transferred to NGM agar plates seeded with foodborne pathogenic bacteria including EDL933 (8.0 × 10^9^ CFU/ml), SL1344 (8.0 × 10^9^ CFU/ml), Newman (8.0 × 10^9^ CFU/ml), and EGD-e (8.0 × 10^9^ CFU/ml) and then incubated at 25°C. For each killing assay, 90 *C. elegans* per treatment were assayed in three plates (30 worms per plate). *C. elegans* were counted daily and transferred to a new plate every 2 days. To determine whether alive or dead, *C. elegans* were gently touched with a platinum wire. The assay was conducted until all *C. elegans* died.

### Worm Size and Locomotive Activity

Worm size and locomotive activity were measured using Wormlab software (MBF Bioscience, USA) as previously reported with slight modifications [19]. Briefly, L4 stage *C. elegans* were fed with OP50 (8.0 × 10^9^ CFU/ml) or 3201 (8.0 × 10^9^ CFU/ml) for 48 h and then transferred to a low peptone NGM plate seeded with OP50. Filming was performed after a 10 min acclimation period. For tracking analysis, the video was taken for 1 min. Width, length, and locomotive activity (peristaltic speed [μm/s]) were measured. Ten worms in each group were measured. The experiments were repeated in triplicate. By using a stereomicroscope, the pharynx pumping rate was measured to estimate food intake. The pumping rate was monitored by counting the pharyngeal contraction for 30 s. At least 10 worms in each group were measured. The experiments were repeated in triplicate.

### RNA Isolation and Transcriptome Analysis

Transcriptome analysis was conducted with a slight modification of the previous study [20]. Briefly, L4 stage *C. elegans* were placed on NGM plates with OP50 (8.0 × 10^9^ CFU/ml) or 3201 (8.0 × 10^9^ CFU/ml). After a 48-h exposure period, total RNA from worms was immediately extracted to evaluate gene expression in the host using TRIZOL reagent (Invitrogen, USA) and purified using an RNeasy Mini Kit (Qiagen, USA) based on the manufacturer’s instructions. For RNA-seq, a TruSeq RNA Sample Prep Kit v2 (Illumina, USA) was used based on the manual, and the cDNA library was made based on the basic protocol provided by Illumina. Libraries were then sequenced on an Illumina NovaSeq 6000 platform with paired end read sequencing (2 × 250 bp). Using Trimmomatic 0.38 [21], the adapter sequence, base quality less than 3 from the ends of the reads, bases not satisfying the window size of 4, and mean quality of 15 were removed. Then to produce trimmed data, reads shorter than 36 bp were removed, and further analysis was performed based on the remaining high-quality reads. The index of the reference genome was generated using the Hisat2 v2.1.0 program (https://daehwankimlab.github.io/hisat2/ accessed on 4 November 2020) [22]. Next, uniquely mapped reads were quantified with Subread/featureCounts version v1.5.1 (http://subread.sourceforge.net/ accessed on 4 November 2020) [23] using ENSEMBL version 82 transcriptome definitions. R package edgeR was used to analyze different expressions between different types of samples in generated data [24]. To define genes that are significantly different in expression, the threshold value of |log2-fold change > 1| and *p* value < 0.05 were used. The Database for Annotation, Visualization, and Integrated Discovery (DAVID) online tool and Cytoscape were used to identify the function of differentially expressed genes.

### Metabolites Extraction and Metabolome Analysis

To measure the change of metabolites, metabolome analysis was performed with a slight modification to a previous study [25]. L4 stage *C. elegans* were fed with OP50 (8.0 × 10^9^ CFU/ml) or 3201 (8.0 × 10^9^ CFU/ml) for 48 h and then washed six times with sterile deionized water to remove bacteria on their body. Then worms were mechanically disrupted using a pestle (Kontes Glass, USA). Each sample was mixed with ice-cold methanol and vortexed vigorously on the ice for 1 min. The vortexed sample was centrifuged at 10,000 ×*g* for 10 min at 4°C. Then the supernatants were filtered with 0.2 μm polyvinylidene fluoride syringe filters (Whatman, UK) and vacuum dried. For gas chromatography–mass spectrometry (GC-MS) analysis, 30 μl of methoxyamine hydrochloride (Sigma–Aldrich, USA) in pyridine (20 mg/ml) was added to each sample to dissolve and then incubated at 30°C for 90 min to derivatize. Then for trimethylsilylation, each sample was added with 50 μl N,O-bis(trimethylsilyl)trifluoroacetamide (Sigma–Aldrich) and incubated at 60°C for 30 min. Lastly, 10 μl of fluoranthene (Sigma–Aldrich) was added.

GC-MS analysis was conducted using a TRACE 1310 Gas Chromatograph (Thermo Fisher Scientific, USA) with an ISQ LT single quadrupole mass spectrometer (Thermo Fisher Scientific), and separation was conducted using a DB-5MS column (60 m × 0.25 mm, 0.25 μm film thickness, Agilent, USA). The oven temperature was held at 50°C for 2 min, constantly increased at 5°C/min, held at 180°C for 8 min, gradually increased at 2.5°C/min to 325°C, and then held for 10 min. The sample was injected at 300°C, and helium was used as the carrier gas, with a flow rate of 1.5 ml/min and a split ratio of 1:60. For GC-MS detection, an electron ionization system with an ionization voltage of 70 eV was used, and the temperature of the ion source was 270°C. The mass scan range was set at 30–450 (m/z), and the acquisition rate was 5 spectra/s. NIST Mass Spectral Search Program (version 2.0; National Institute of Standards and Technology [NIST], USA) was used to identify detected metabolites. Further analyses were performed using MetaboAnalyst 6.0.

### Statistics

*C. elegans* lifespan and killing assay data were analyzed through the Kaplan–Meier method and graphed using SigmaPlot 12.0 (Systat Software Inc.). Other data were statistically analyzed with Prism 9 (GraphPad Software, USA). Statistical significance was considered when *p* values below 0.05 (*), 0.01 (**), 0.001 (***), and 0.0001 (****). The graphs are presented as mean ± standard error of the mean (SEM).

### Data Availability

All the data needed to assess the conclusions of the paper are included in the manuscript and deposited in the NCBI SRA database under the Bioproject number PRJNA1092403. Additional data can be obtained from the authors upon request.

## Results

### Evaluation of the Adhesive Ability of *L. rhamnosus* IDCC 3201 in *C. elegans*

**Fig. 1:**
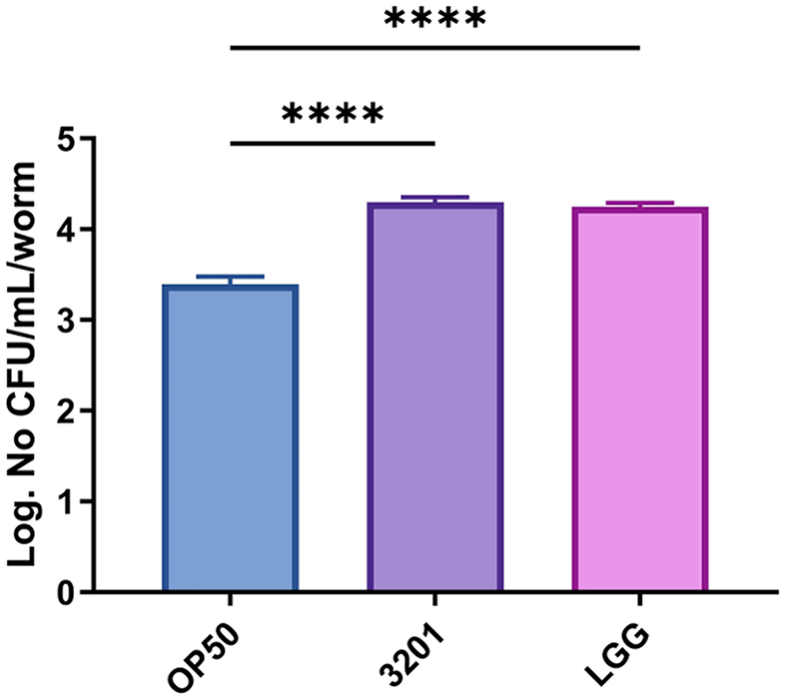
Evaluation of the adhesive ability of *Lacticaseibacillus rhamnosus* IDCC 3201 in *C. elegans*. The adhesive ability of OP50, 3201, or LGG in *C. elegans* strain *fer-15;fem-1* after 48 h conditioning period. OP50, *E. coli*; 3201, *L. rhamnosus* IDCC 3201; LGG, *L. rhamnosus* GG. Statistical analysis was performed using one-way ANOVA, and statistical significance was considered when the *p* values were below 0.05 (*), 0.01 (**), 0.001 (***), and 0.0001 (****). Statistics compared with 3201: *p* < 0.0001 and *p* = 0.3699 for OP50 and LGG, respectively. Data are expressed as means ± SEM.

We first evaluated the adhesive ability of probiotic candidate bacteria *L. rhamnosus* IDCC 3201 (3201) in *C. elegans*. We confirmed the adhesive ability after 48 h exposure period on OP50, 3201, or LGG. The result showed that 3201 exhibited significantly higher adhesive ability compared with OP50 (*p* < 0.0001), and there was no significant difference compared with LGG (*p* = 0.3699) (Fig. 1). These results suggested that 3201 has strong adhesive ability in *C. elegans*.

### Evaluation of Longevity and Immune Response of *L. rhamnosus* IDCC 3201 Using *C. elegans*

**Fig. 2:**
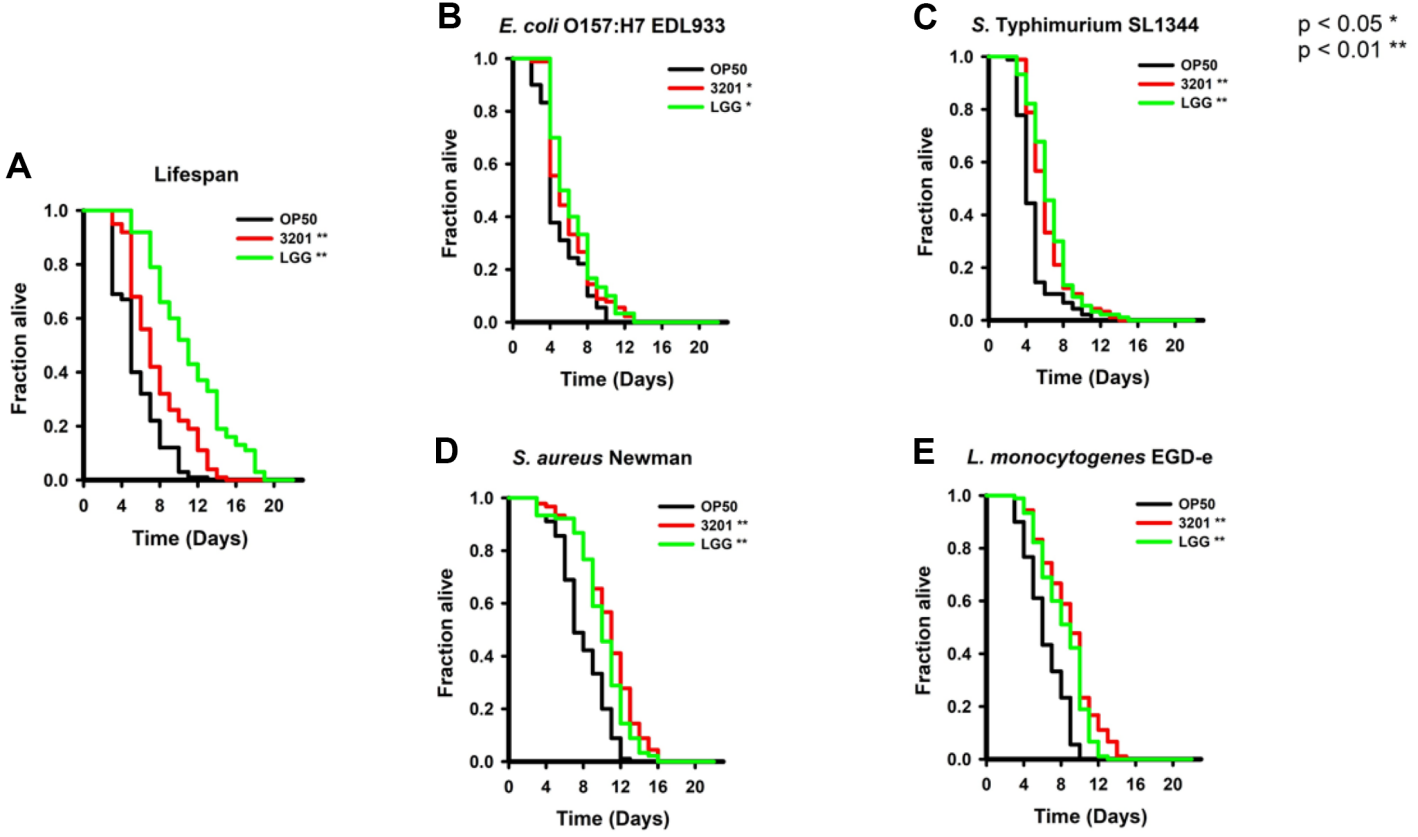
Evaluation of longevity and immune response of *Lacticaseibacillus rhamnosus* IDCC 3201 using *C. elegans*. The lifespan of *C. elegans* strain *fer-15;fem-1* using OP50, 3201, and LGG. The killing assay of *C. elegans* strain fer- 15;fem-1 preconditioned with OP50, 3201, or LGG for 48 h then infected with each of four foodborne pathogenic bacteria (two of each gram-negative and positive bacteria). (**A**) Lifespan assay. (**B**) Killing assay using *Escherichia coli* O157:H7 EDL933. (**C**) Killing assay using *Salmonella* Typhimurium SL1344. (**D**) Killing assay using *Staphylococcus aureus* Newman. (**E**) Killing assay using *Listeria monocytogenes* EGD-e. OP50, *E. coli*; 3201, *L. rhamnosus*; LGG, *L. rhamnosus*. Statistical analysis was performed using the Kaplan–Meier method, and differences were considered significant when the *p* value was below 0.05 (*) and 0.01 (**). Survival statistic in lifespan assay compared with 3201: *p* = 0.0000 and *p* = 0.0000 for OP50 and LGG, respectively. Survival statistics in killing assay compared with 3201: *Escherichia coli* O157:H7 EDL933, *p* = 0.0114 and *p* = 0.5001 for OP50 and LGG, respectively; *Salmonella* Typhimurium SL1344, *p* = 0.0000 and *p* = 0.2732 for OP50 and LGG, respectively; *Staphylococcus aureus* Newman, *p* = 0.0000 and *p* = 0.0000 for OP50 and LGG, respectively; *Listeria monocytogenes* EGD-e, *p* = 0.0000 and *p* = 0.0496 for OP50 and LGG, respectively. Data are expressed as means ± *SEM*.

We examined whether 3201 can improve the lifespan of *C. elegans*. The *C. elegans* were treated with OP50, 3201, or LGG. The group fed with OP50 was referred to as OP50, the group fed with 3201 was referred to as 3201, and the group fed with LGG was referred to as LGG. The result showed that the 3201 group showed a significantly extended lifespan compared with the OP50 group which was used as a negative control in this study (*p* = 0.0000)(Fig. 2A).

Subsequently, we investigated the effect of 3201 on the host’s immune response against four different kinds of foodborne pathogenic bacteria. *C. elegans* were preconditioned with OP50, 3201, or LGG for 48 h and then transferred to plates seeded with four pathogens. The results showed that the 3201 group showed increased resistance against gram-negative pathogens compared with the OP50 group (*p* = 0.0115 for EDL933 and *p* = 0.0000 for SL1344). In addition, the 3201 group showed no significant difference from the LGG group (*p* = 0.5001 for EDL933 and *p* = 0.2732 for SL1344) (Fig. 2B and 2C). The study using gram-positive pathogens showed that the 3201 group showed enhanced immune response against gram-positive pathogens compared with the OP50 group (*p* = 0.0000 for Newman and *p* = 0.0000 for EGD-e). Interestingly, the 3201 group showed improved immune response compared with the LGG group (*p* = 0.0000 for Newman and *p* = 0.0496 for EGD-e)(Fig. 2D and 2E). Taken together, these results indicated that 3201 can improve the lifespan and resistance of *C. elegans* against both gram-negative and gram-positive pathogenic bacteria.

### Evaluation of Phenotypic Change of *C. elegans* after Exposure to *L. rhamnosus* IDCC 3201

**Fig. 3:**
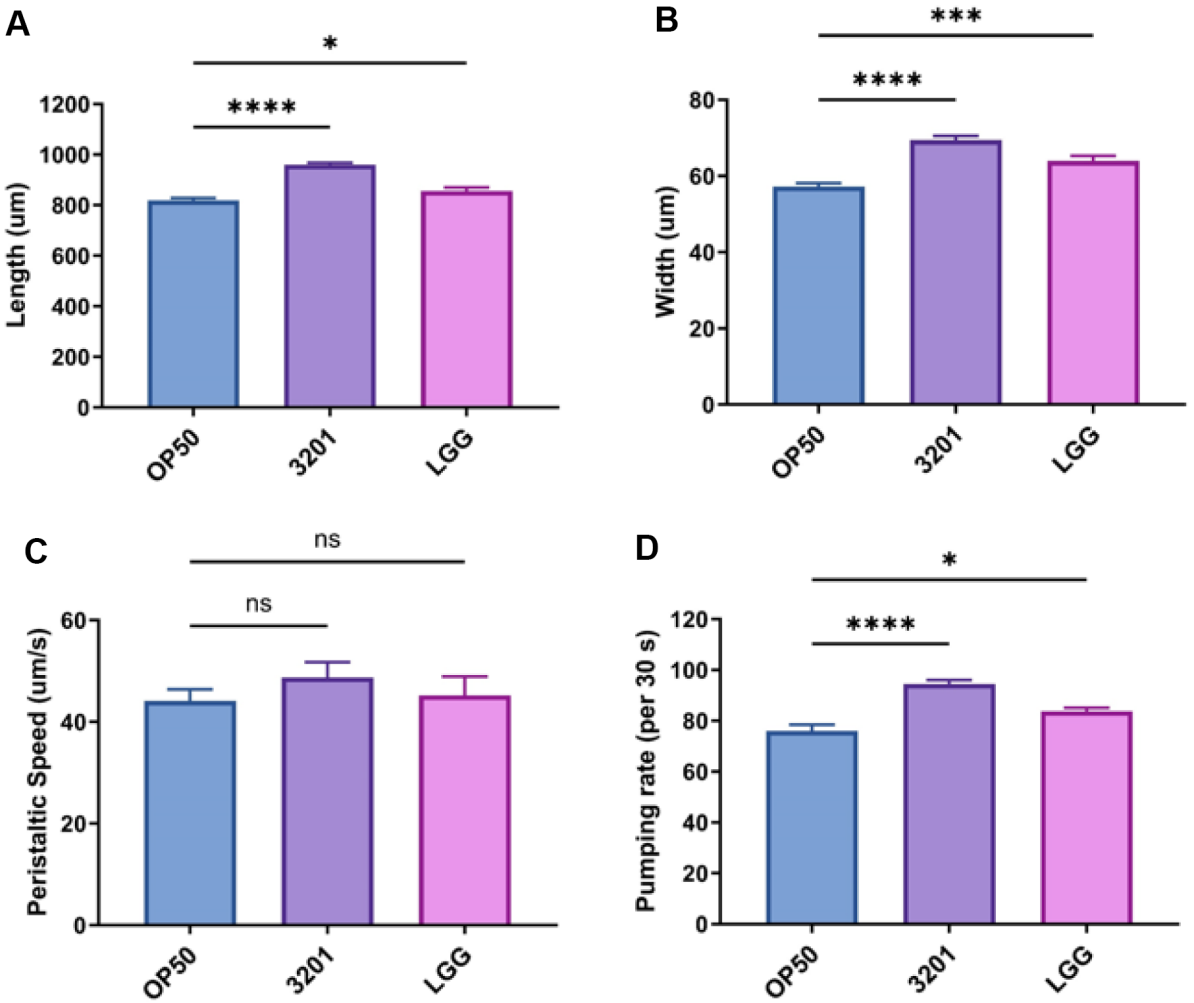
Evaluation of phenotypic change of *C. elegans* after exposure to *Lacticaseibacillus rhamnosus* IDCC 3201. Worm size and locomotive activity of *C. elegans* strain *fer-15;fem-1* after 48 h exposure period with OP50, 3201, or LGG. (**A**) Length, (**B**) Width, (**C**) Peristaltic speed, and (**D**) Pumping rate. OP50, *E. coli*; 3201, *L. rhamnosus*; LGG, *L. rhamnosus*. Statistical analysis was performed using one-way ANOVA, and statistical significance was considered when the *p* values were below 0.05 (*), 0.01 (**), 0.001 (***), and 0.0001 (****). Statistics compared with 3201: length, *p* < 0.0001 and *p* < 0.0001 for OP50 and LGG, respectively; width, *p* < 0.0001 and *p* = 0.0028 for OP50 and LGG, respectively; peristaltic speed, *p* = 0.5366 and *p* = 0.6957 for OP50 and LGG, respectively; pumping rate, *p* < 0.0001 and *p* = 0.0002 for OP50 and LGG, respectively. Data are expressed as means ± *SEM*.

We measured the body size to evaluate whether feeding 3201 changed the phenotype of worms. The body size was measured after feeding with OP50, 3201, or LGG for 48 h. The results showed that the 3201 group demonstrated a significant increase in both length and width compared with both the OP50 group (*p* < 0.0001 for length and *p* < 0.0001 for width) and the LGG group (*p* < 0.0001 for length and *p* < 0.0028 for width) (Fig. 3A and 3B). The peristaltic speed, which refers to the activity of worms, showed no difference between the 3201 and OP50 groups (*p* = 0.5366) (Fig. 3C). In the pumping rate, which refers to the food intake, the 3201 group exhibited a significant increase compared with both the OP50 and LGG groups (*p* < 0.0001 for OP50 and *p* = 0.0002 for LGG)(Fig. 3D). Taken together, 3201 enhanced the body size, activity, and pumping rate of *C. elegans*.

### Evaluation of Longevity and Immune Response of *L. rhamnosus* IDCC 3201 Using *C. elegans*

We examined whether 3201 can improve the lifespan of *C. elegans*. The *C. elegans* were treated with OP50, 3201, or LGG. The group fed with OP50 was referred to as OP50, the group fed with 3201 was referred to as 3201, and the group fed with LGG was referred to as LGG. The result showed that the 3201 group showed a significantly extended lifespan compared with the OP50 group which was used as a negative control in this study (*p* = 0.0000)(Fig. 2A).

Subsequently, we investigated the effect of 3201 on the host’s immune response against four different kinds of foodborne pathogenic bacteria. *C. elegans* were preconditioned with OP50, 3201, or LGG for 48 h and then transferred to plates seeded with four pathogens. The results showed that the 3201 group showed increased resistance against gram-negative pathogens compared with the OP50 group (*p* = 0.0115 for EDL933 and *p* = 0.0000 for SL1344). In addition, the 3201 group showed no significant difference from the LGG group (*p* = 0.5001 for EDL933 and *p* = 0.2732 for SL1344) (Fig. 2B and 2C). The study using gram-positive pathogens showed that the 3201 group showed enhanced immune response against gram-positive pathogens compared with the OP50 group (*p* = 0.0000 for Newman and *p* = 0.0000 for EGD-e). Interestingly, the 3201 group showed improved immune response compared with the LGG group (*p* = 0.0000 for Newman and *p* = 0.0496 for EGD-e)(Fig. 2D and 2E). Taken together, these results indicated that 3201 can improve the lifespan and resistance of *C. elegans* against both gram-negative and gram-positive pathogenic bacteria.

### Evaluation of Phenotypic Change of *C. elegans* after Exposure to *L. rhamnosus* IDCC 3201

We measured the body size to evaluate whether feeding 3201 changed the phenotype of worms. The body size was measured after feeding with OP50, 3201, or LGG for 48 h. The results showed that the 3201 group demonstrated a significant increase in both length and width compared with both the OP50 group (*p* < 0.0001 for length and *p* < 0.0001 for width) and the LGG group (*p* < 0.0001 for length and *p* < 0.0028 for width) (Fig. 3A and 3B). The peristaltic speed, which refers to the activity of worms, showed no difference between the 3201 and OP50 groups (*p* = 0.5366) (Fig. 3C). In the pumping rate, which refers to the food intake, the 3201 group exhibited a significant increase compared with both the OP50 and LGG groups (*p* < 0.0001 for OP50 and *p* = 0.0002 for LGG)(Fig. 3D). Taken together, 3201 enhanced the body size, activity, and pumping rate of *C. elegans*.

### Transcriptomic Analysis of *C. elegans* after Exposure to *L. rhamnosus* IDCC 3201

**Fig. 4:**
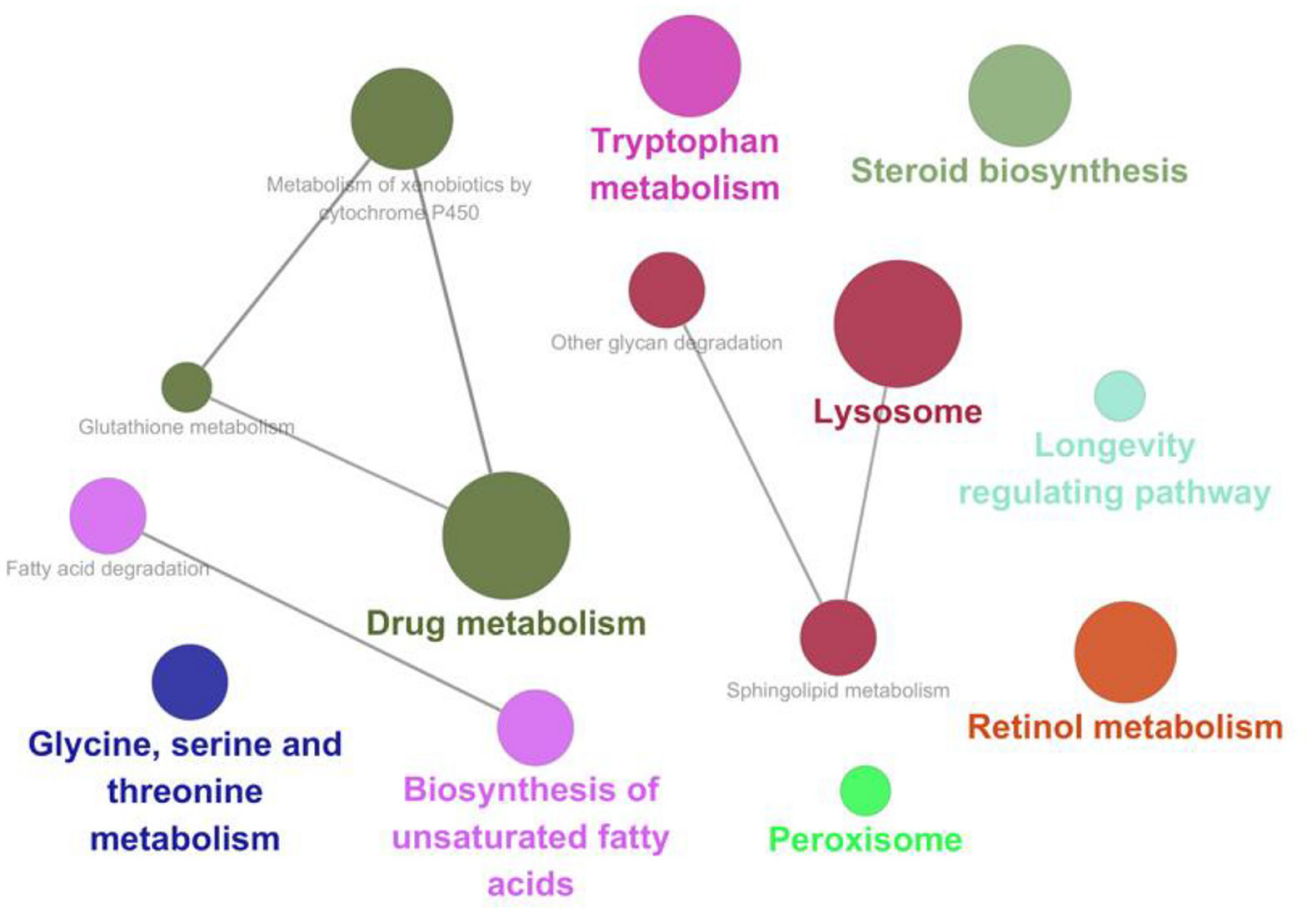
Transcriptomic analysis of *C. elegans* after exposure to *Lacticaseibacillus rhamnosus* IDCC 3201. The identification of KEGG pathways related to genes significantly upregulated by more than 3-fold in *C. elegans* when fed with 3201 compared with OP50. We utilized Cytoscape for the analysis.

We conducted a transcriptome analysis to evaluate the altered mechanism through feeding 3201 compared with OP50. We selected genes that showed a significant upregulation of more than a 2-fold change in *C. elegans* fed with 3201 compared with those fed with OP50. These selected genes were then used to examine the upregulated pathways through DAVID. The top 10 pathways that are most closely associated with significantly upregulated genes are shown in Table 1. In line with previous killing assay results, pathways related to innate immune response, defense response to gram-positive bacteria, and defense response to gram-negative bacteria were upregulated by feeding 3201. Within the innate immune response pathway, genes such as *clec-41*, *clec-62*, *clec-86*, *clec-186*, and *clec-187* (C-type lectin-related) and *lys-1*, *lys-2*, *lys-5*, *lys-7*, and *lys-8* (Lysozyme-related) were upregulated with 3201 feeding (Table 2). To identify the KEGG pathways upregulated by feeding 3201, genes showing significant upregulation of more than a 3-fold change were analyzed using Cytoscape. The results revealed several significantly upregulated pathways with 3201, namely drug metabolism, biosynthesis of unsaturated fatty acids, glycine serine and threonine metabolism, tryptophan metabolism, lysosome, peroxisome, steroid biosynthesis, longevity regulating pathway, and retinol metabolism (Fig. 4). Overall, these findings suggested that 3201 may enhance the innate immune response against foodborne pathogenic bacteria.

### Metabolomic Analysis of *C. elegans* after Exposure to *L. rhamnosus* IDCC 3201

**Fig. 5:**
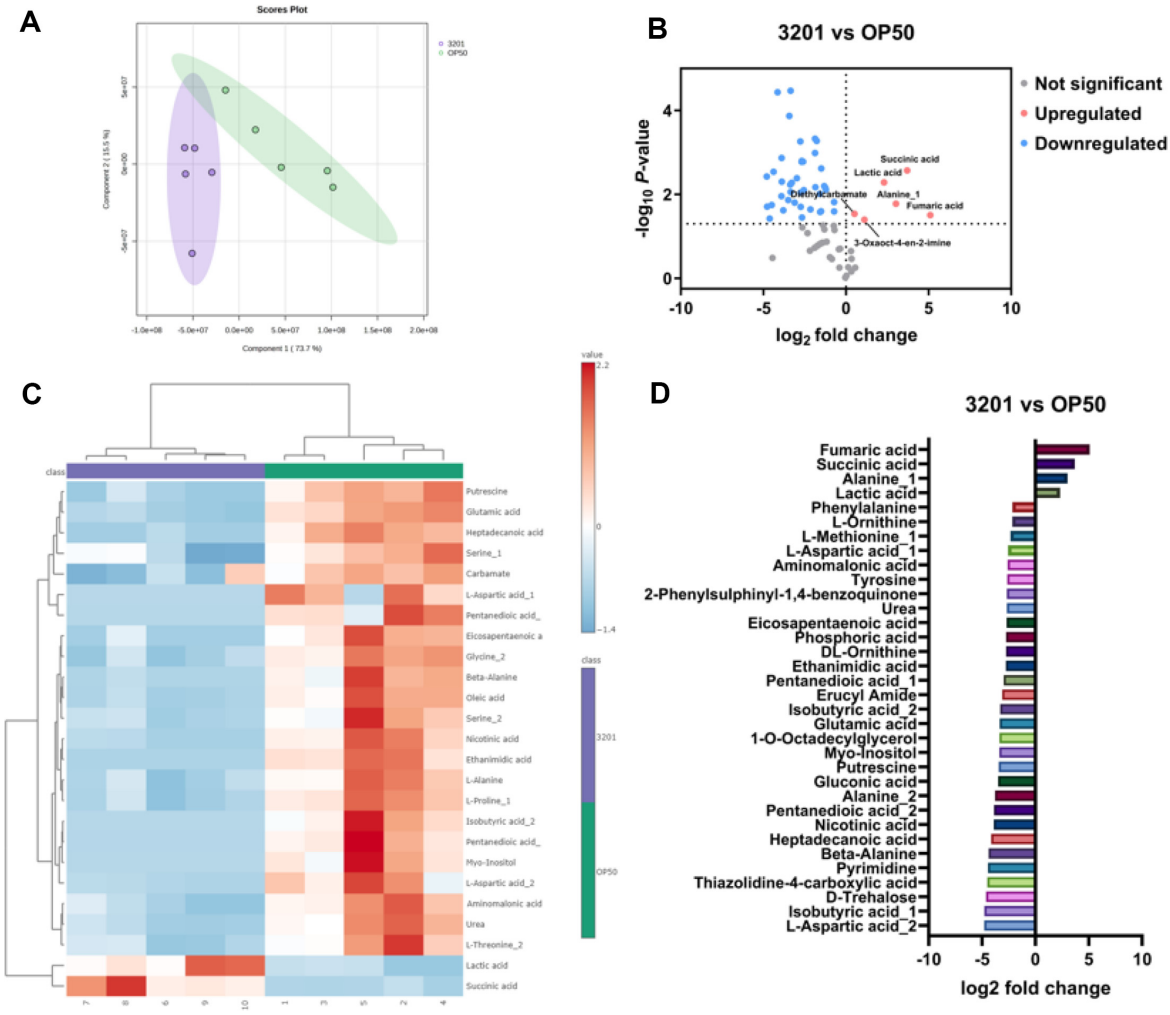
Metabolomic analysis of *C. elegans* after exposure to *Lacticaseibacillus rhamnosus* IDCC 3201. Change of metabolites composition in *C. elegans* strain *fer-15;fem-1* after 48 h exposure period with OP50 or 3201. (**A**) PLS-DA. (**B**) Volcano plot. (**C**) Top 25 enriched heatmap. (**D**) The quantitative graph using the metabolites which changed more than 4.0 folds.

The metabolomic analysis was performed to assess the impact of 3201 on the metabolite composition of *C. elegans*. The result showed a distinct clustering between worms fed with 3201 and OP50 (Fig. 5A). The volcano plot was performed to illustrate statistical significance (*p* value) and fold change of individual metabolites. Notably, six metabolites including fumaric acid, succinic acid, alanine, lactic acid, 3-oxaoct-4-en-2-imine, and diethylcarbamate were significantly upregulated in *C. elegans* treated with 3201 compared with those fed with OP50 (Fig. 5B). Further analysis through the top 25 enriched heatmap data revealed consistent elevation of succinic acid and lactic acid in all samples of *C. elegans* fed with 3201 compared with OP50 (Fig. 5C). The quantitative graph was created using a fold change threshold of 4 for the metabolites. The result showed that four metabolites, namely fumaric acid, succinic acid, alanine, and lactic acid, were quantitatively increased in *C. elegans* fed with 3201 compared with OP50 (Fig. 5D). In summary, 3201 induced a shift in metabolite composition, specifically leading to an increase in certain metabolites, such as lactic acid.

## Discussion

According to the previous study, probiotics are known to exert their beneficial effects on the host by adhering to host epithelial cells, modulating the host’s immune response, and producing specific metabolites [26]. *Lactobacillus* spp. is one of the most widely used probiotics due to its health-enhancing properties [27]. *Lactobacilli* are gram-positive bacteria and produce lactic acid through carbohydrate fermentation, which is known as an antimicrobial compound [28, 29]. In previous studies, some strains of *Lactobacillus rhamnosus* (recently reclassified as *Lacticaseibacillus rhamnosus*) have shown positive effects on the host [30, 31]. Therefore, we aimed to evaluate the probiotic properties of *Lacticaseibacillus rhamnosus* IDCC 3201 (3201) as a candidate probiotic bacterium using the *C. elegans* surrogate animal model, which is commonly used in vivo model to study the interaction of bacteria and host [25].

According to a previous study, the adhesive ability to the host’s gastrointestinal is one of the classical selection criteria for potential probiotic bacteria. This adhesive capability can lead to colonization, which can help improve immunomodulatory effects and stimulate the gut barrier and metabolic functions [32]. Our study revealed that 3201 exhibits a significantly higher adhesive ability than OP50 and shows no significant difference compared with LGG. In lifespan assay, 3201 significantly improved the longevity of *C. elegans* compared with OP50. These findings align with the previous study that *L. rhamnosus* Lcr35 can adhere to the *C. elegans* gut, resulting in beneficial effects such as prolonged longevity and inhibition of the colonization of pathogenic bacteria [33]. In addition, the previous study reported that feeding *C. elegans* with *L. rhamnosus* NBRC14710 significantly improved its lifespan. In the killing assay, preconditioning with 3201 significantly enhanced the immune response of *C. elegans* against foodborne pathogenic bacteria compared with OP50. These results parallel the previous studies demonstrating that preconditioning with probiotic *Lactobacillus* spp. can augment the immune response of *C. elegans* against both gram-negative and gram-positive pathogenic bacteria [16-18].

According to previous studies, the quality of food and some types of bacteria are known to affect the phenotype of worms such as showing better growth performance [34-36]. Therefore, we sought to assess the impact of feeding 3201 on various phenotypic traits. Measurements of body size, peristaltic speed, and pumping rate were employed as indicators of potential changes in the worms’ phenotype. The results revealed a significant increase in both length and width when *C. elegans* were fed with 3201 compared with those fed with OP50 and LGG. In terms of pumping rate, 3201 demonstrated a significant increase compared with OP50 and LGG. Collectively, these results demonstrated that feeding 3201 induces a phenotypic change in worms and enhances growth performance.

Our previous findings revealed that preconditioning with 3201 enhanced resistances against both gram-negative and gram-positive foodborne pathogens. Therefore, we speculated that 3201 may modulate specific gene expression related to the immune response. Transcriptome analysis using genes that significantly increased more than 2-fold in *C. elegans* fed with 3201 compared with OP50 revealed that the innate immune response was the most relevant pathway. Among the genes associated with the innate immune response, C-type lectin-related genes (*clec-41*, *clec-86*, *clec-62*, *clec-186*, and *clec-187*) and lysozyme-related genes (*lys-1*, *lys-2*, *lys-5*, *lys-7*, and *lys-8*) showed significant upregulation in *C. elegans* fed with 3201. C-type lectin-like domain (CTLD) proteins, encoded by clec genes, play diverse roles, including immune response in vertebrates. In *C. elegans*, these genes encode both CTLD proteins and others similar to CTLD proteins in vertebrates, functioning with the immune system [37]. The previous study has demonstrated that clec genes exhibit a pathogen-specific response, with specific pathogen infections leading to the upregulation of specific clec genes [38]. However, the specific role of each clec gene remains to be elucidated. According to a previous study, the inactivation of *clec-41* increased susceptibility to *Bacillus thuringiensis* MYBt18247 infection, highlighting its crucial role in resistance against pathogenic bacteria. In addition, *clec-41* protein demonstrated antimicrobial activity against *E. coli* and *B. thuringiensis* Bt247 by binding to bacteria [37]. These results indicated that *clec-41* is a crucial gene for resistance against pathogenic bacteria. In agreement with a previous study, *clec-41* was the second significantly upregulated C-type lectin-related gene after feeding 3201 for 48 h compared with feeding OP50. Another gene, *clec-86*, was shown to be essential in defending against gram-positive pathogenic bacteria *Microbacterium nematophilum*, emphasizing its importance in the defense response [39]. In agreement with the previous study, *clec-86* was the most significantly upregulated C-type lectin-related gene after preconditioning with 3201 compared with OP50. Furthermore, the previous study revealed that infection with the gram-negative pathogenic bacteria *Pseudomonas aeruiginosa* upregulated *clec* genes, including *clec-41*, *clec-62*, *clec-86*, *clec-186*, and *clec-187* [38]. Interestingly, these were the same genes significantly upregulated by feeding with 3201 in our study, which suggests that the upregulation of clec genes through preconditioning with 3201 may contribute to an improved immune response against pathogenic bacteria. Lysozymes, recognized as antimicrobial factors, play a role in breaking up microbial cell walls in *C. elegans* intestine and provide resistance against pathogenic bacteria [40, 41]. A previous study suggested that lysozyme-related genes, particularly *lys-1*, *lys-3*, and *lys-7* contribute significantly to the defense response in *C. elegans* [38]. Consistent with the previous study, the expression of *lys-1* and *lys-7* genes was significantly upregulated in *C. elegans* fed with 3201 compared with those fed with OP50. We speculated that increased expression of C-type lectin-related genes and lysozyme-related induced by feeding with 3201 may contribute to an enhanced immune response against pathogenic bacteria.

The metabolome analysis revealed increased levels of fumaric acid and succinic acid, metabolites of the citric acid cycle, in *C. elegans* fed with 3201. The previous studies showed that increased citric acid cycles are related to extended lifespan [42, 43]. Consistent with our findings, supplying fumaric acid in a previous study resulted in an increased lifespan for *C. elegans* [44]. Increasing the citric acid cycle is known as the increasing NAD/NADH ratio, which may be crucial for the mechanism of the lifespan of *C. elegans* [45]. In this study, increased fumaric acid by feeding 3201 may have increased mitochondrial respiration and enhanced NAD/NADH ratio, which may have extended the lifespan of *C. elegans*. In addition, lactic acid was significantly elevated in *C. elegans* fed with 3201 compared with those fed with OP50. This result aligns with existing knowledge that certain *Lactobacillus* spp. including *L. rhamnosus* have the capability to produce antimicrobial compounds such as lactic acid, bacteriocin, and H_2_O_2_ [46]. These compounds can inhibit the growth of pathogenic bacteria and fortify the defense mechanisms of *C. elegans* against pathogens [47-49]. In summary, the increased levels of fumaric acid and succinic acid by feeding 3201 may have played a role in extending the lifespan of *C. elegans*, potentially through their impact on the citric acid cycle. Simultaneously, the increased level of lactic acid by feeding 3201 may have contributed to a robust defense response against pathogenic bacteria.

In conclusion, our study focused on evaluating the probiotic properties of *L. rhamnosus* IDCC 3201 (3201) using *C. elegans* as the surrogate animal model. The results demonstrated that 3201 exhibited superior adhesive capabilities compared with OP50 and performed similarly to LGG, highlighting its potential for effective gastrointestinal colonization. Furthermore, 3201 significantly extended the lifespan of *C. elegans*, enhanced its resistance against foodborne pathogenic bacteria, and improved growth performance. Transcriptome analysis unveiled a significant upregulation of genes associated with the innate immune response, particularly C-type lectin-related and lysozyme-related genes, indicating a potential molecular mechanism for the observed immune enhancement. Metabolome analysis revealed elevated levels of fumaric acid, succinic acid, and lactic acid in *C. elegans* fed with 3201, suggesting a potential mechanism for extending lifespan and fortifying defense against pathogens. In summary, our study demonstrated that *L. rhamnosus* IDCC 3201 is a promising probiotic candidate with potential applications in promoting host health and immunity.

## Figures and Tables

**Table 1:** Transcriptomic analysis of *C. elegans* after exposure to *Lacticaseibacillus rhamnosus* IDCC 3201.

Term	Gene count	%	*p* value
Innate immune response	75	4.8	0.000
Glutathione metabolic process	18	1.1	0.000
Peptidoglycan catabolic process	8	0.5	0.000
Cell wall macromolecule catabolic process	9	0.6	0.000
Defense response to gram-positive bacterium	17	1.1	0.000
Lipid metabolic process	32	2	0.000
Proteolysis	42	2.7	0.000
Defense response to gram-negative bacterium	19	1.2	0.000
Dephosphorylation	13	0.8	0.000
Fatty acid beta-oxidation using acyl-CoA oxidase	5	0.3	0.000

Top 10 pathways closely associated with genes significantly upregulated more than 2.0 folds in *C. elegans* strain *fer-15;fem-1* following 48 h of exposure to *L. rhamnosus* 3201 compared with *E. coli* OP50.

**Table 2:** Transcriptomic analysis of *C. elegans* after exposure to *Lacticaseibacillus rhamnosus* IDCC 3201.

Group and gene	Gene number	Fold change	*p* value	Description
C-type lectin-related				
*clec-41*	CELE_B0365.6	8.443320	0.000	C-type lectin
*clec-62*	CELE_F35C5.5	2.348795	0.000	C-type lectin
*clec-86*	CELE_C54D1.2	9.677637	0.000	C-type lectin
*clec-186*	CELE_ZK896.7	6.833677	0.000	C-type lectin
*clec-187*	CELE_ZK896.6	4.984468	0.000	C-type lectin
Lysozyme-related				
*lys-1*	CELE_Y22F5A.4	3.182571	0.000	Lysozyme
*lys-2*	CELE_Y22F5A.5	8.078466	0.000	Lysozyme
*lys-5*	CELE_F22A3.6	12.360469	0.000	Lysozyme
*lys-7*	CELE_C02A12.4	5.542044	0.000	Lysozyme
*lys-8*	CELE_C17G10.5	2.706994	0.000	Lysozyme

List of genes that were significantly upregulated by more than 2.0 folds in *C. elegans* when fed with *L. rhamnosus* 3201 compared with *E. coli* OP50 and associated with the innate immune response pathway.
